# NONCOMBAT AND COMBAT MILITARY SERVICE EXPERIENCES, HEARING DIFFICULTY, AND DIFFICULTY REMEMBERING/CONCENTRATING

**DOI:** 10.1093/geroni/igad104.0163

**Published:** 2023-12-21

**Authors:** Andrew London, Scott Landes, Janet Wilmoth

**Affiliations:** Syracuse University, Maxwell School of Citizenship and Public Affairs, Syracuse, New York, United States; Syracuse University, Syracuse, New York, United States; Syracuse University, Syracuse, New York, United States

## Abstract

Hearing difficulty is widespread among U.S. veterans. Research documents a strong association between hearing difficulty and a range of cognitive outcomes. Most research that examines cognitive outcomes in older adults does not distinguish veterans from non-veterans or non-combat from combat veterans. We use data on Sample Adults aged 50+ from the 2021 National Health Interview Survey to examine: the associations between military service experiences (non-veteran, non-combat veteran, combat veteran) and difficulty remembering/concentrating (none, some, a lot/cannot do at all) net of sociodemographic controls; the extent to which observed associations are mediated by hearing difficulty (none, some, a lot/cannot do at all); whether military service experiences moderate the association between hearing loss and difficulty remembering and concentrating. This latter hypothesis is motivated by the notion that service-related hearing loss may occur earlier in the life course and therefore be more detrimental to cognitive outcomes. Analyses are weighted; standard errors are adjusted for the complex sampling design. Preliminary results from multivariable ordinal logistic regression models that include sociodemographic controls indicate that: non-combat and combat veterans have significantly more difficulty remembering/concentrating than non-veterans; the associations between non-combat and combat military service experiences and increased difficulty remembering/concentrating are reduced 20% when hearing difficulty is added to the model, but remain statistically significant; hearing difficulty is strongly associated with difficulty remembering/concentrating; and military service experiences moderate the associations between hearing difficulty and difficulty remembering/concentrating. For example, the highest level of hearing difficulty is more strongly associated with difficulty remembering/concentrating among combat veterans than non-combat veterans.

